# Validation of the Chinese Version of the Coronary Artery Disease Education Questionnaire – Short Version: A Tool to Evaluate Knowledge of Cardiac Rehabilitation Components

**DOI:** 10.5334/gh.912

**Published:** 2021-02-24

**Authors:** Lamei Yang, Shilan Luo, Siyu Yang, Gabriela Lima de Melo Ghisi, Xiaoli Yan, Mengying Tian, Yunmei Wu

**Affiliations:** 1 Department of Geriatric Cardiology, The Second Affiliated Hospital of Chongqing Medical University, Chongqing, CN; 2 Second clinical college, Chongqing Medical University, CN; 3 Cardiovascular Prevention Rehabilitation Program, Toronto Rehabilitation, University Health Network, Toronto, CA; 4 Health Management Center, The First Affiliated Hospital of Chongqing Medical University, CN

**Keywords:** cardiovascular disease, education, validation, cardiac rehabilitation, knowledge

## Abstract

**Background:**

Patient education is the first step in implementing a cardiac rehabilitation (CR) program and a powerful tool for promoting behavioral changes in cardiac patients. In China, the clinical workload is so heavy that a short and reliable tool for assessing disease-related knowledge is needed for targeted patient education.

**Objective:**

The aim of this study was to translate, adapt and validate the Chinese version of the Coronary Artery Disease Education Questionnaire – Short Version (CADE-Q SV).

**Methods:**

The CADE-Q SV was translated to simplified Chinese and culturally adapted to the Chinese context. The translated version was reviewed by a committee of seven experts in cardiovascular disease, and the content validity of the questionnaire was established. The psychometric properties of the questionnaire were analyzed considering the responses of 240 CR patients. The Kuder-Richardson-20 (KR-20) coefficient and Cronbach’s alpha were used to assess internal consistency. The intraclass correlation coefficient (ICC) was used to assess test-retest reliability. The criterion-related validity was evaluated by determining whether there were differences in the total scores of patients with different educational levels. Confirmatory factor analysis (CFA) was used to assess the factor structure.

**Results:**

Three items from the original version were adapted to reflect Chinese culture. The content validity index was 0.94. The KR-20 score was 0.856. All ICC values were > 0.70. The knowledge scores of patients with different educational levels were significantly different, indicating that the criterion-related validity of the Chinese CADE-Q-SV was acceptable. CFA validated the five-factor structure of the Chinese CADE-Q-SV.

**Conclusion:**

The Chinese CADE-Q SV questionnaire has good reliability and validity. This short, efficient tool can be completed quickly, assess disease-related knowledge in cardiovascular patients and serve as a reference for individualized patient education in China. It can also be used to evaluate the effectiveness of CR-related patient education interventions.

## Introduction

The incidence of cardiovascular disease (CVD) is still increasing worldwide, especially in low- and middle-income countries, and CVD remains the leading cause of death in China and worldwide [1,2]. According to relevant reports, CVD is likely to cause 23.3 million deaths by 2030 [3]. In 2019, the World Health Organization renewed its call for high-quality prevention and treatment of chronic diseases as global development goals [4]. The US Department of Health and Human Services also proposed the ‘Million Hearts’ plan in 2012 and updated the plan 2017, aiming to prevent 1 million cardiovascular events within five years [5,6]. In China, the burden of CVD is also increasing and has become a major public health problem. The ‘Summary of the 2018 Report on Cardiovascular Diseases in China’ shows that the number of cases of CVD in China is 290 million; of these, coronary artery disease (CAD) accounts for 11 million cases, and the mortality rate of CAD is higher than that of tumors and other diseases in rural and urban areas in China. The trend has been increasing annually, so prevention and treatment methods are urgent [7].

For patients with CAD, secondary prevention strategies, such as cardiac rehabilitation (CR), have been shown to reduce the incidence of acute cardiovascular events, reduce mortality, and improve quality of life by promoting behavioral changes [8,9]. However, CR is underutilized throughout this process; typically, only 30% to 50% of cardiovascular patients in Europe participate in outpatient CR, and only 13.9% of those complete the eight weeks CR program in the UK [10,11,12]. Due to the later emergence of CR in China than in Europe, patients’ lack of knowledge about the disease, and patients’ lack of knowledge about the benefits of CR, the rate of participation in CR in China is relatively low [13,14]. Therefore, disease-related patient education is extremely important, and questionnaires to evaluate knowledge components of CR are also urgently needed.

CVD patients can benefit from long-term adherence to a CR program, and studies have suggested that patient education is significantly correlated with CR participation, and education and exercise are equally important in cardiovascular patients [15]. Patient education is an intervention that promotes self-management in clinical populations, specifically among patients with CVD [16,17]. Studies have shown that education of patient with CVD is associated with improvements in both patient self-management behaviors and quality of life and reductions in both medical costs and the rate of recurrence of acute events [16,18,19,20,21,22]. Therefore, it is necessary to improve the long-term participation rate in CR programs in patients with CVD by improving patient education. The first step in patient education is to assess the current level of patient knowledge about CVD.

Ghisi et al. developed the Coronary Artery Disease Education Questionnaire (CADE-Q) in 2010 and a second version (CADE-Q II) in 2015 [23,24]. Many studies have used this tool in different settings; it has been used in a number of randomized controlled studies, and it has been validated in Portuguese and in other languages [22,25,26,27,28,29]. Although both versions have good reliability and validity, the CADE-Q lacked a detailed evaluation of nutritional and psychosocial risks, which are core components of CR knowledge [24]. On this basis, the CADE-Q II was developed. Since both tools take approximately 20 minutes to complete, Ghisi et al. aimed to improve the efficiency of the questionnaire; accordingly, they developed the Coronary Artery Disease Education Questionnaire-Short Version (CADE-Q SV) in 2016 to assess disease-related knowledge among CR patients [30]. Under the heavy clinical workload in China [31], the CADE-Q SV could effectively save time for medical staff. Moreover, healthcare professionals can use the results of questionnaire surveys to develop individualized education for patients; questionnaires can also be used to evaluate the effects of educational interventions to optimize patient education [23].

The aim of this study was to translate, acculturate, and psychometrically validate the Chinese version of the CADE-Q SV.

## Method

### Design and procedure

In this study, the questionnaire was translated from English to Simplified Chinese through forward translation, back translation and cultural adaptation. The translated version was psychometrically tested in a cross-sectional study. The study was approved by the Institutional Review Board of The Second Affiliated Hospital of Chongqing Medical University. The data were collected from October 2019 to July 2020.

The use of the CADE-Q SV was authorized by Ghisi, the author of the original questionnaire, and suggestions by Guillemin et al. were adopted for translation and cultural adjustment [32]. 1) The forward translation process was as follows: the English version of the questionnaire was independently translated by two researchers who were proficient in both Chinese and English, one of whom was a cardiovascular doctor with overseas study experience in the United States and the other who was a postgraduate nursing student at the University of Edinburgh. 2): The proofreading process was as follows: a discussion group compared the two Chinese translations, selected the appropriate translation, and finally synthesized the two translation results into one version. 3) The back translation process was as follows: a mock version was translated from Chinese into English by a college English professor and a medical doctor. To ensure the quality of the back translation, neither translator had any contact with the original questionnaire and did not know they were performing back-translation work. Then, a detailed comparison was made between the back-translated English questionnaire and the original English questionnaire to determine the sentences with a semantic consistency rate less than 70%. The identified statements were retranslated repeatedly until the semantic consistency rate reached 90%. Finally, we invited the original author to review the back-translated version to ensure there was no meaningful difference from the original English version. 4) The cultural adaptation process was as follows: an expert team (including three nursing professors, two experienced associate chief nurses in geriatric cardiology, and two chief physicians in CR research and clinical work) independently reviewed the original, proofread and back-translated version of the questionnaire, and evaluated its content validity. The discussion group collated and analyzed the experts’ opinions and chose the most appropriate Chinese expressions.

Subsequently, the Chinese CADE-Q SV was pilot tested with 30 Chinese CR patients, and in-depth interviews were conducted to assess the patients’ understanding of the items word by word. Timely modifications were made to items with vague meanings and unintelligibility. After completing the above steps, the Chinese CADE-Q SV was finalized.

Third, the Chinese version was administered to CR patients who were recruited by convenience sampling. CVD patients were referred to the CR center by cardiology or geriatric outpatient physicians or residents to participate in CR on a voluntary basis. Patients were approached at any time during the program. The CR program included participation by doctors, nurses, CR therapists, psychologists, and dietitians. Patient education and exercise were carried out simultaneously, and patient education was mainly performed by nurses. CR patients received collective patient education two to three times a month, and patient education was also provided at each step of the CR program.

### Participants

The participants were recruited from a CR center, and a Geriatric Cardiology Department of grade III A hospital in Chongqing, China. The inclusion criteria were as follows: 1) patients with confirmed heart disease or multiple cardiovascular risk factors; 2) patients undergoing CR after evaluation and recommendation by medical personnel; and 3) patients who were able to understand the questionnaire content, provide informed consent and participate voluntarily. The exclusion criteria were as follows: 1) patients who were illiterate; and 2) patients who were unable to complete the questionnaire for any reason.

All CR participants who met the inclusion criteria signed a consent form before enrollment in this research. Then, questionnaires were used to collect relevant patient data. The characteristics of CR participants were analyzed according to sex, age, marital status, living area, family income, educational level, clinical risk factors and so on. The required sample size was five to 10 times the number of items, assuming a 20% rate of invalid questionnaires; therefore, a total of 240 questionnaires were distributed and completed, with a response rate of 100%.

### Measures: the CADE-Q SV scale

The CADE-Q SV is a questionnaire used to assess knowledge of CR components, with a total of 20 questions and five domains: medical condition, risk factors, exercise, nutrition, and psychosocial risk. Each domain has four questions. Patients answer only true/false/I do not know, with one point for correct answers, no points for wrong answers or the response ‘I do not know.’ The total possible score is 20 points, and the higher the score, the more knowledge the patient has about the disease [33]. The original version of the tool was developed in English and psychometrically tested in Canadian CR participants [30]. Since then, the questionnaire has also been shown to have good reliability and validity among CAD patients who speak Brazilian Portuguese and other languages [34,35]. Therefore, the questionnaire can also be used to develop educational interventions involving disease-related knowledge for cardiovascular patients, not just for patients in CR programs.

### Data analysis

First, the expert team evaluated the content validity of the questionnaire. Second, the Kuder-Richardson-20 (KR-20) coefficient was calculated to assess the internal consistency of the whole questionnaire, and Cronbach’s alpha was calculated to assess the internal consistency of each factor. Third, the intraclass correlation coefficient (ICC) was calculated by readministering the questionnaire after two weeks in 30 patients to assess the test-retest reliability. A patient’s education level may affect their disease-related knowledge level, so this study compared the scores of patients with different education levels to assess the criterion validity in this study [26,28]. Fourth, confirmatory factor analysis (CFA) was used to examine the factor structure in this study.

The maximum likelihood (ML) method was applied for parameter estimations using the chi-square to df (x^2^/df) ratio, root mean square residual (RMR), the goodness-of-fit index (GFI), comparative fit index (CFI), and root mean square error of approximation (RMSEA) to evaluate whether the approximation error model was good or bad [36]. The X^2^/df was ≤3, GFI was >0.90, CFI was >0.90, RMSEA was ≤0.08, and RMR was < 0.05 meeting the study requirements [37].

Finally, the frequency and percentage of counting data were described. The mean ± standard deviation (x̄ ± SD) was used to describe measurement data that conformed to a normal distribution; and quartiles (P25, P75) were used to represent measurement data that did not conform to a normal distribution. In the univariate analysis, the t test was used for dichotomous variables with a normal distribution, and the t’ test was used for those with uneven variance. The nonparametric Mann-Whitney U test (to calculate the Z value) was used for the bivariate variables with a nonnormal distribution. For multiple categorical variables, if the data were normally distributed and the variance was homogeneous, ANOVA was performed (to calculate the F value); if the data were skewed or the variance was uneven, the nonparametric Kruskal-Wallis H test (to calculate the H value) was used. SPSS 26.0 was used for all statistical analyses [38].

## Results

### Translation, back translation, and cultural adaptation

In the process of back translation, problems such as missing words and mistranslation of the items were resolved. Semantic equivalence between the back-translated version and the original version was achieved.

Through discussion among the individuals on the expert panel, three items were modified. Because Chinese people prefer to eat foods such as kimchi, the phrase ‘prepared, processed foods’ in item nine was modified to ‘pickled foods.’ The statement ‘sodium in the diet to less than 2000 mg per day’ in item 12 was revised to ‘salt in the diet to less than 6 g per day’ to align with the Chinese Dietary Guidelines [39]. Because most Chinese people were not familiar with trans fats and hydrogenated vegetable oils, item 14 was modified to read, ‘Trans fats are unhealthy fats that are often found in fried or baked foods.’ Thirty CR patients participated in the pilot test, and they took approximately 10 minutes to complete the questionnaire. According to the feedback, there were no unclear or ambiguous items in the translated version.

### Psychometric validation

The content validity index of the items ranged from 0.857 to 1.00, and the content validity index of the whole questionnaire was 0.94. Because the minimum requirement for this value is 0.7, [40] the results indicated that the questionnaire had acceptable content validity. The KR-20 assessed the internal consistency of the entire questionnaire, and all factors were reliable (Cronbach’s alpha values of each domain were between 0.747 and 0.817). The KR-20 and Cronbach’s Alpha values of each item met the requirements. The test-retest reliability for each item was assessed by the ICC, and the ICCs for all items were greater than the recommended minimum values. Items with an ICC lower than 0.7 were excluded from the analysis (Table 1) [41]. The results also showed that the knowledge scores of patients with different educational levels were different (P < 0.001); specifically, the more educated the patients were, the higher their knowledge scores (P < 0.001), indicating that the criterion-related validity of the Chinese CADE-Q SV was acceptable (Table 2).

**Table 1:** The score of CADE-Q SV and Cronbach’s alpha coefficients.

Area	Items	Score mean ± SD	ICC	Item total score correlations	Cronbach’s alpha coefficient per domain	Mean Score Per area

Medical	1	0.49 ± 0.50	0.76	0.421**	0.747	1.78 ± 1.50
	3	0.47 ± 0.50	0.77	0.548**		
	6	0.42 ± 0.49	0.76	0.509**		
	11	0.41 ± 0.49	0.79	0.578**		
Risk Factors	2	0.66 ± 0.47	0.92	0.565**	0.749	2.56 ± 1.45
	12	0.65 ± 0.48	0.83	0.489**		
	16	0.57 ± 0.50	0.76	0.556**		
	18	0.67 ± 0.47	0.92	0.575**		
Exercise	4	0.78 ± 0.42	0.84	0.465**	0.780	3.05 ± 1.31
	8	0.86 ± 0.35	0.79	0.419**		
	13	0.73 ± 0.45	0.81	0.619**		
	17	0.69 ± 0.46	0.79	0.672**		
Diet	5	0.84 ± 0.37	0.76	0.405**	0.743	3.28 ± 1.15
	9	0.87 ± 0.34	0.84	0.426**		
	14	0.73 ± 0.45	0.84	0.676**		
	20	0.84 ± 0.37	0.79	0.333**		
Psychological Risk	7	0.70 ± 0.46	0.91	0.454**	0.817	2.48 ± 1.54
	10	0.67 ± 0.47	0.76	0.466**		
	15	0.47 ± 0.50	0.77	0.544**		
	19	0.64 ± 0.48	0.81	0.591**		
Total		13.15 ± 4.70	–	1	0.856	–

*Note*: ** P < 0.001. SD-standard deviation; ICC-intraclass correlation coefficient; *Abbreviation*: CADE-Q SV, Coronary Artery Disease Education Questionnaire – Short Version.

**Table 2:** Demographic characteristics of participants (n = 240).

Variable		N(%)	Scores(mean ± SD) or M(P_25_, P_75_)	H/Z	P–value

Age, years(mean ± SD)		65.14 ± 11.76			
Age dichotomous	Less than 65 years old	96(40.0)	14.00(11.00,18.00)	–3.055^a^	0.002
	65 years old or older	144(60.0)	12.38 ± 4.73		
Gender	Male	135(56.3)	14.00(10.00,18.00)	–1.787^a^	0.074
	Female	105(43.7)	12.56 ± 4.60		
Marital status	Married	218(90.8)	13.00(10.00,18.00)	5.609^b^	0.061
	Single	4(1.7)	11.75 ± 5.68		
	Divorced/widowed	18(7.5)	10.72 ± 4.97		
Living area	City	163(67.9)	15.00(12.00,18.00)	–7.387^a^	<0.001
	Countryside	77(32.1)	9.95 ± 3.81		
Monthly family income	Less than three thousand yuan	25(10.4)	9.00(6.00,18.50)	5.891^b^	0.117
	Between three and five thousand yuan	103(42.9)	13.00(10.00,17.00)		
	Between five and 10 thousand yuan	109(45.4)	14.00(11.00,18.00)		
	More than 10 thousand yuan	3(1.3)	12.00 ± 3.00		
Education background	Primary school and below	51(21.3)	8.00(6.00,11.00)	101.896^b^	<0.001
	Middle school	57(23.8)	11.86 ± 4.07		
	High school	61(25.4)	13.00(12.00,17.00)		
	Bachelor degree and above	71(29.5)	18.00(15.00,19.00)		
Comorbidity	No	17(7.1)	7.00(6.00,9.00)	70.522^b^	<0.001
	With one comorbidity	77(32.1)	11.10 ± 4.20		
	With two comorbidity	110(45.8)	14.00(11.00,17.00)		
	With three comorbidity	36(15.0)	19.00(17.50,20.00)		
History of CVD	Less than one year	7(2.9)	13.14 ± 5.52	2.267^b^	0.322
	Between one and five years	119(49.6)	12.71 ± 4.57		
	More than five years	114(47.5)	14.00(10.00,18.00)		
Smoking	yes	67(28.0)	11.99 ± 4.50	–2.439^a^	0.015
	no	173(72.0)	14.00(10.00,18.00)		
Alcoholic behavior	yes	44(18.3)	14.00(11.00,18.00)	–1.579^a^	0.114
	no	196(81.7)	13.00(10.00,17.00)		
Myocardial infarction		73(30.4)	13.00(10.00,18.00)	–0.286^a^	0.775
PCI		89(37.1)	12.00(9.00,18.00)	–0.650^a^	0.516
Duration in CR (mean ± SD)		1.73 ± 1.14			
	1 month	78(32.5)	12.50(9.00,17.00)	15.736^b^	0.008
	2 months	91(38.0)	12.00(10.00,16.50)			
	3 months	44(18.3)	13.57 ± 4.47		
	4 months	15(6.2)	16.60 ± 2.67		
	5 months	6(2.5)	16.33 ± 3.33		
	6 months	6(2.5)	15.17 ± 1.94		

*Note*: Comorbidity, such as hypertension, diabetes, and hyperlipidemia; three thousand yuan corresponds to USD$ 423.22; PCI, Percutaneous Transluminal Coronary Intervention. ^a^ = Z-value, ^b^ = H-value.

Regarding factor analysis, the results from the Kaiser-Meyer-Olkin index (KMO = 0.836) and Bartlett’s sphericity tests (x^2^ = 1795.87, p < 0.001) indicated that the data were suitable for factor analysis, CFA was used to test the five-factor model established in the original validation [22]. The results showed that the model has good data fitting (x^2^/df = 1.766; RMSEA = 0.056; CFI = 0.930; GFI = 0.900; RMR = 0.011). The factor loading coefficients of the model ranged from 0.458 to 0.919, and all were within the acceptable level. Therefore, the five-factor Chinese version of the CADE-Q SV was acceptable (Table 3).

**Table 3:** Factor loadings from confirmatory factor analysis.

Area	Items	Unstandardized estimate	Standard error	P-value	Standardized estimate

Medical	1	1.000	0.000	999.000	0.515
	3	1.403	0.201	***	0.724
	6	1.252	0.188	***	0.654
	11	1.337	0.194	***	0.704
Risk Factors	2	1.000	0.000	999.000	0.648
	12	0.979	0.124	***	0.624
	16	1.075	0.131	***	0.659
	18	1.039	0.125	***	0.671
Exercise	4	1.000	0.000	999.000	0.513
	8	0.871	0.125	***	0.521
	13	1.565	0.209	***	0.732
	17	1.898	0.241	***	0.858
Diet	5	1.000	0.000	999.000	0.458
	9	1.025	0.152	***	0.513
	14	2.390	0.366	***	0.919
	20	1.003	0.189	***	0.464
Psychological Risk	7	1.000	0.000	999.000	0.682
	10	1.107	0.118	***	0.727
	15	1.170	0.125	***	0.723
	19	1.188	0.122	***	0.761

*Note*: * P < 0.001.

### Characteristics of the study participants

A total of 240 eligible subjects participated in this study. The characteristics of the participants are described in Table 2. Overall, 240 (100%) cardiovascular ambulatory patients completed the Chinese version of the CADE-Q SV; 135 respondents (56.3%) were male, 105 respondents (43.7%) were female, and the mean age was 65.14 ± 11.76 years old.

### Cardiovascular patients’ knowledge about their condition

Table 1 shows the means and standard deviations of the total score of the Chinese CADE-Q SV as well as the scores for each factor and each item. The following items had the highest scores (i.e., had the highest number of correct answers): ‘the content of sodium salt is usually high in pickled foods,’ ‘warming up before exercise can slowly increase heart rate and reduce the risk of angina pectoris,’ and ‘eating more meat and dairy products is a good way to increase dietary fiber intake.’ The following items had the lowest scores: ‘“statin” medications (such as atorvastatin and simvastatin) limit how much cholesterol your body absorbs from food,’ ‘antiplatelet drugs such as aspirin (ASA) are important to reduce the “stickiness” of blood platelets and make it easier for blood to flow through coronary arteries and coronary stents,’ and ‘“angina” is a chest pain or discomfort, during rest or physical activity, as well as pain in the arms, back and/or neck.’ The domain with the highest level of knowledge was diet, and the domain with the lowest level of knowledge was medical condition. Scores were examined to determine which demographic characteristics influenced the participants’ knowledge levels. The results showed that living area (P < 0.001), educational background (P < 0.001), and comorbidities (P < 0.001) significantly impacted the score of the questionnaire. In addition, younger participants (i.e., less than 65 years old) scored significantly higher in knowledge than those participants who were 65 years old or older.

## Discussion

Education should be a component of CR programs, as it is a powerful tool to promote behavioral change and increase the quality of life of cardiac patients. During CR, assessing a patient’s level of knowledge is an important step in educational intervention.

In this study, strict reliability and validity tests were conducted to evaluate the Chinese CADE-Q SV questionnaire, and the results showed that the questionnaire had good reliability and validity. The Chinese CADE-Q SV was developed after three items from the original version were modified and adapted to be consistent with Chinese culture. The pilot study showed that these changes were appropriate. The KR20 and Cronbach’s alpha coefficients indicated that the results of this study were basically consistent with the original questionnaire, which has good internal consistency and reliability. Studies have shown a link between a patient’s level of education and disease-related knowledge [30]. The criterion-related validity of the Chinese CADE-Q SV was verified by comparing patients with different levels of education. The results showed that the higher the education level was, the higher the questionnaire score was, indicating that this questionnaire has an acceptable criterion-related validity. Therefore, it can be used to assess the disease-related knowledge among CR patients in China.

The knowledge score of the respondents in the questionnaire was moderate (13.15 ± 4.70), similar to that in a study conducted in Brazil [34], but lower than that in a study conducted in Canada (16.50 ± 2.15) [30]. This difference indicated the lack of patient education in China [42]. In the future, medical staff should not only strengthen the patient education of CR patients, but also the education of non-CR patients to improve CR knowledge. Chinese CR patients had higher knowledge scores in nutrition and exercise but lower knowledge scores in medical conditions and psychosocial risks. The results were similar to those of study by Ghisi et al. [30], which indicated that healthcare providers should place increased emphasis on providing CR patients with knowledge of medical conditions and psychosocial risk factors.

There were also significant differences in the knowledge scores among people with different characteristics. The results showed that patients who lived in urban areas, had higher education levers, did not smoke, had two or more comorbidities, and had a long duration of CR had a better understanding of disease-related knowledge than those who did not [29,43]. Compared with patients in rural areas, patients in urban areas are closer to CR clinics and CR centers, making it more convenient for them to access CR resources [43]. Therefore, China should strengthen the construction of CR centers, delegate CR to primary-level medical institutions and train primary-level medical professionals to assist rural patients. Similar to a previous study, patients with two or three comorbidities had higher questionnaire scores than those with less than two comorbidities [30]. The possible reason for this difference is that these comorbidities reinforce the patient’s awareness of the disease. Medical staff should strengthen publicity and education for patients with complications to improve patients’ awareness of the disease, avoid deterioration of the disease, and promote patient recovery. The longer the CR period was, the higher the knowledge score was, which may be the reason for the increase in the frequency of disease-related patient education.

In the future, further studies should be conducted to determine the psychometric characteristics of the CADE-Q SV in China. First, with respect to the potential strategies to educate cardiovascular patients, it should be further determined whether the questionnaire is effective in judging the effectiveness of CR-related patient education. Second, the Portuguese version of the CADE-Q SV has been shown to have good reliability and validity in non-CR patients. In the future, the efficacy of the CADE-Q SV questionnaire in evaluating non-CR patients can also be explored in China. Third, in a previous study, exploratory factor analysis (EFA) revealed that the English version of the CADE-Q SV had five factors [30]. The CFA model fitting indexes in this study show that the five-factor model fits well and that all factor loadings reached acceptable levels, indicating that the construct validity of the Chinese CADE-Q SV is good. While the original author used EFA to establish the five-factor questionnaire model, EFA was not used again for factor analysis in this study. In future research, EFA can be incorporated into the research method to further analyze the structure of factors.

Finally, the type of sample and the fact that participants were recruited from only one site also limits the generalizability of this study.

## Conclusions

In summary, the Chinese CADE-Q SV was shown to have strong psychometric properties, and this study introduced an efficient tool for assessing the knowledge of CR patients in China. It is hoped that this tool will help healthcare providers develop personalized CR plans in CR programs. This study also provides reference for medical staff to use the questionnaire in clinical and research settings for the assessment of CR-related knowledge and the development of patient education interventions.

## Additional File

The additional file for this article can be found as follows:

10.5334/gh.912.s1 Original data. CADE-Q SV scores, demographic information, and clinical data from 240 CR patients.
